# MYC is Sufficient to Generate Mid-Life High-Grade Serous Ovarian and Uterine Serous Carcinomas in a p53-R270H Mouse Model

**DOI:** 10.1158/2767-9764.CRC-24-0144

**Published:** 2024-09-26

**Authors:** Alexandra Blackman, Amy C. Rees, Robert R. Bowers, Christian M. Jones, Silvia G. Vaena, Madison A. Clark, Shelby Carter, Evan D. Villamor, Della Evans, Anthony J. Emanuel, George Fullbright, Matthew S. O’Malley, Richard L. Carpenter, David T. Long, Laura S. Spruill, Martin J. Romeo, Brian C. Orr, Kristi L. Helke, Joe R. Delaney

**Affiliations:** 1 Department of Obstetrics and Gynecology, Medical University of South Carolina, Charleston, South Carolina.; 2 Department of Biochemistry and Molecular Biology, Medical University of South Carolina, Charleston, South Carolina.; 3 Hollings Cancer Center, Medical University of South Carolina, Charleston, South Carolina.; 4 Department of Pathology and Laboratory Medicine, Medical University of South Carolina, Charleston, South Carolina.; 5 Department of Medical Sciences, Indiana University Bloomington, Bloomington, Indiana.; 6 Department of Biochemistry and Molecular Biology, Indiana University Bloomington, Bloomington, Indiana.; 7 Department of Comparative Medicine, Medical University of South Carolina, Charleston, South Carolina.

## Abstract

Genetically engineered mouse models (GEMM) have fundamentally changed how ovarian cancer etiology, early detection, and treatment are understood. *MYC*, an oncogene, is amongst the most amplified genes in high-grade serous ovarian cancer (HGSOC), but it has not previously been utilized to drive HGSOC GEMMs. We coupled *Myc* and dominant-negative mutant p53-R270H with a fallopian tube epithelium (FTE)-specific promoter *Ovgp1* to generate a new GEMM of HGSOC. Female mice developed lethal cancer at an average of 14.5 months. Histopathologic examination of mice revealed HGSOC characteristics, including nuclear p53 and nuclear MYC in clusters of cells within the FTE and ovarian surface epithelium. Unexpectedly, nuclear p53 and MYC clustered cell expression was also identified in the uterine luminal epithelium, possibly from intraepithelial metastasis from the FTE. Extracted tumor cells exhibited strong loss of heterozygosity at the p53 locus, leaving the mutant allele. Copy-number alterations in these cancer cells were prevalent, disrupting a large fraction of genes. Transcriptome profiles most closely matched human HGSOC and serous endometrial cancer. Taken together, these results demonstrate that the *Myc* and *Trp53-R270H* transgenes were able to recapitulate many phenotypic hallmarks of HGSOC through the utilization of strictly human-mimetic genetic hallmarks of HGSOC. This new mouse model enables further exploration of ovarian cancer pathogenesis, particularly in the 50% of HGSOC which lack homology-directed repair mutations. Histologic and transcriptomic findings are consistent with the hypothesis that uterine serous cancer may originate from the FTE.

**Significance:**

Mouse models using transgenes which generate spontaneous cancers are essential tools to examine the etiology of human diseases. Here, the first Myc-driven spontaneous model is described as a valid HGSOC model. Surprisingly, aspects of uterine serous carcinoma were also observed in this model.

## Introduction

Gynecologic oncology has only recently been able to take advantage of the targeted therapies generated over the last decade despite there being great need. Ovarian cancer is the fifth most common cause of cancer-related death in women, and uterine cancer is the sixth. Ovarian cancer deaths are dominated by the high-grade serous ovarian cancer (HGSOC) subtype. Uterine cancer, overall, is less lethal despite higher incidence rates, yet the serous carcinoma subtype accounts for 40% of endometrial cancer deaths (1). Uterine cancer incidence and mortality have increased due to a disproportionate increase in type 2 or nonendometrioid subtypes, including serous carcinoma (2).

Recent targeted therapies in HGSOC involve PARP inhibitors (PARPi), which best benefit patients with *BRCA1* or *BRCA2* tumor mutations or markers of homologous repair deficiency: half of patients in sum (3). Uterine cancers have shown promise in large phase III trials of immunotherapies, particularly in mismatch repair–deficient cases (4, 5), but only a small subset of patients with serous carcinoma is mismatch repair–deficient. Unfortunately, few targeted drugs have shown clinical benefit for HGSOC with normal homologous repair or in recurrent tumors which restore homologous repair after PARPi therapy. Similarly, serous endometrial cancer has few targetable alterations and thus few drugs in development (6). Serous endometrial cancer and HGSOC share similar disease etiology, such as postmenopausal disease presentation and peritoneal metastasis, and similar molecular characteristics, such as low mutation rates, high copy-number alterations (CNA), mutation in p53, and few other driver mutations.

Genetically engineered mouse models (GEMM) have fundamentally changed how ovarian cancer etiology and treatment is understood. Phylogenetic assessments of HGSOC compared primary tumors dissected from an ovary and metastases, which demonstrated that the earliest mutations often originated in serous tubal intraepithelial carcinoma (STIC; ref. 7). However, this study and other studies have shown that cells can arise first on the ovarian surface epithelium (OSE) or other origin sites, metastasize to the fallopian tube epithelium (FTE) and develop STIC, and finally develop into HGSOC (8–10). A recent pair of GEMMs indicated that OSE-originating cancer cells are phenotypically distinct from fallopian fimbriae–originating cancer cells despite identical drivers (9). *Lgr5* was used to drive OSE gene deletion, whereas *Pax8* was used to drive FTE gene deletion. Previous research established the *Ovgp1* model of oviductal transgene expression, in which *Ovgp1*-mediated expression control was exclusive to the FTE (11, 12).

These previous mouse models revolutionized the field but have some important limitations. Most models utilized homozygous deletions of multiple tumor suppressors to generate tumors, which is not often, if ever, found in human disease. We previously investigated aneuploidy with or without a heterozygous deletion of the autophagy gene *Becn1* in the *Amhr2*-SV40-T-antigen model (13, 14). These viral proteins are known to disrupt p53 function and RB1 function, amongst other proteins (15, 16). However, few aneuploid events were observed relative to human disease (14), similar to other GEMM findings (17). Previous HGSOC mouse models utilize *BRCA* deletions, *PTEN* deletions, or viral oncogenes. The field is left without a genetically representative model for HGSOC lacking *BRCA1/2* or other homologous repair alterations (∼50% of human tumors) or HGSOC lacking a *PTEN* deletion (94% of human tumors). Here, we introduce a new mouse model of ovarian cancer driven by p53 mutation and MYC under the *Ovgp1* promoter. Evidence of hallmarks of HGSOC and uterine serous cancer are described.

## Materials and Methods

### Transgenic OvTrpMyc mouse model creation

Transgenic mice were generated by the Taconic/Cyagen Transgenic Animal Center (Cyagen Biosciences Inc.). Briefly, *Trp53* (NM_001127233.1) containing an R270H mutation and *Myc* (NM_001177352.1) were knocked into the endogenous Trp53 locus in a reverse orientation using CRISPR-Cas9–mediated gene editing. A construct with 985 bp of the murine *Ovgp1* promoter, including its proximal epigenetic regulatory peak (Supplementary Fig. S1A; ref. 18), was cloned upstream of the murine *Trp53*-coding sequence containing a consensus Kozak sequence and an R270H mutation (CGT to CAT mutation in codon 270) without a stop codon. A P2A self-cleaving peptide sequence precedes the murine *Myc*-coding sequence which contains a 3× hemagglutinin (HA) C-terminal tag and is followed by a bovine growth hormone polyadenylation sequence. Homology arms were supplied by bacterial artificial chromosomes RP23-240I24 and RP23-243M15 (19). The complete transgenic sequence is supplied as Supplementary File 1.

### Mouse husbandry, genotyping, and loss of heterozygosity

All mouse procedures were approved by the Medical University of South Carolina’s Institutional Animal Care and Use Committee, protocol #01305, prior to use. Animals were housed in individual vented cages in rooms with 12:12 light/dark cycle and were fed ad libitum (Purina Pro-Lab 5V75). Founder mice were generated in the C57BL/6Tc background and backcrossed to C57BL/6J (RRID: IMSR_JAX:000664) for experimentation. Litters were always formed by crossing a transgenic male with a wild-type (WT) C57BL/6 mother obtained externally (Jackson Laboratories, #000664). For genotyping, ∼2-mm sections of mice tails or ear punches were lysed at 55°C overnight in 350 μL of buffer containing 100 mmol/L Tris-HCl pH 8.8, 5 mmol/L ethylenediaminetetraacetic acid (EDTA) pH 8.0, 0.2% SDS, 200 mmol/L NaCl, and 100 μg/mL proteinase K (VWR, 97062-670). After briefly centrifuging to spin down debris, DNA in 300 μL of lysate was precipitated by adding 1 mL of 100% ethanol. DNA was then pelleted by centrifugation at 16,000 *g* for 30 minutes at 4°C. The supernatant was discarded, and the pellet was washed with 70% ethanol. After centrifugation at 16,000 *g* for 20 minutes at 4°C, the pellet was resuspended in 50 μL of tris-EDTA (TE: 10 mmol/L Tris-HCl, 0.2 mmol/L EDTA; pH 7.5). Finally, the tubes were placed in a heat block at 55°C for 2 hours with lids open to evaporate the remaining ethanol. Two PCRs per mouse were used to distinguish mutant and WT mice. Both PCRs used the same reverse primer (GTGAGATTTCATTGTAGGTGCCAG), and the forward primers were either specific to the knockin construct (CAGTTGAGATGGAGGAGACCTAGG) or the WT *Trp53* allele (AGCCTGTTGAGCTTCACCCC). For loss of heterozygosity (LOH) studies, identical genotyping PCRs were performed, using genomic DNA extracted from *ex vivo* grown cells via Invitrogen PureLink Genomic DNA Mini Kit (#K182001).

Matings between male heterozygous OvTrpMyc and female C57BL/6J mice exhibited typical Mendelian inheritance. Of 585 offspring mice, 307 (52%) were male and 278 (48%) were female and 305 (52%) were WT and 280 (48%) were transgenic. Male mice were 47% transgenic and 53% WT; female mice were 49% transgenic and 51% WT. None of these differences were statistically different from the 1:1 ratios predicted by Mendelian segregation.

### Longitudinal study of lethal tumor burden

All mice intended to be studied for end-of-life criteria were carefully monitored for signs of tumor burden starting at 2 months of age. The mice were monitored twice weekly for (i) indications of ascites by swollen abdomen, (ii) palpable tumors present externally on the mouse, and (iii) weight loss. Euthanasia was performed when the mice had a palpable tumor of >1 cm, any ascites was observed to increase over 2 days, gait was impaired, or weight loss surpassed 15% of the maximal observed weight. Mice that were found dead for unknown reasons were censored and not included in the Kaplan–Meier curve, and histology was never performed on these mice for quality control reasons. Comparison genotypes were previously published by other studies for p53+/− (20) and Pax8-driven models (17).

### Histopathology

The mice had uterine horns and attached fallopian tubes, ovaries, and fat pads surgically removed. Tissues were fixed overnight in 10% normal buffered formalin (VWR 10790-714) and then transferred to 70% ethanol for 24 hours. The Shandon Citadel 2000 Processor (Thermo Fisher Scientific) was used to process tissues on a 4-hour cycle with the following reagent cycles: 70% ethanol followed by two changes of 95% ethanol, three changes of 100% ethanol, one cycle in a 1:1 solution of ethanol and xylene, three changes in xylene, and two changes in paraffin. During the embedding process, ovaries with a portion of the fallopian tube were laid down flat, and the remaining sections of the uterus were stood perpendicular for cross-sections, averaging 3 to 4 cross-sections of the uterus. After the paraffin hardened, the tissue block was cut in 20-μm sections until full sections of the ovaries and fallopian tubes were visible. Then, 5-μm sections were collected onto positively charged slides, air-dried at room temperature overnight, and heat-fixed at 60°C for 1 hour before hematoxylin and eosin (H&E) staining or IHC. IHC utilized Leica Bond III for automated processing. Antibodies used included rabbit anti-PAX8 antibody (Proteintech 10336-1-AP, RRID: AB_2236705) at a 1:2,000 dilution, rabbit anti-p53 antibody (Leica Biosystems NCL-p53-CM5p, RRID: AB_563933) at a 1:5,000 dilution, anti–c-MYC antibody (Abcam ab32072, RRID: AB_731658) at a 1:500 dilution, anti–pan keratin antibody (Roche 760-2595, RRID: AB_2941938) at its provided ready-to-use dilution, and rabbit anti-HA antibody (Cell Signaling Technology 3724, RRID: AB_1549585) at a 1:5,000 dilution. Sections were imaged on a BioTek Lionheart FX microscope, and images were processed using Gen5 and ImageJ software (RRID: SCR_003070). Mice without visible tumors were necropsied, and H&E slides were assessed by a veterinary pathologist for Supplementary Fig. S5 and by a gynecologic pathologist for Supplementary Fig. S6.

### *Ex vivo* tumor cell culturing conditions

A cohort of mice was used to isolate cells from ovaries, uteruses, or visible tumors. The mice were euthanized and immediately dissected for cell extraction. Ovaries and fallopian tubes were excised from fat pads and uteruses. The mid-section of the uterus was used for uterine cell extraction. Visible tumors were excised from surrounding tissues. All tissues were first rinsed once in sterile PBS, and the PBS was then aspirated. Single, ethanol-sterilized razor blades were then used to mince 2-mm sections of tissue in a tissue culture hood. Thoroughly minced tissue sections were then pipetted using a P1000 pipette into a 6-well tissue culture dish, again dispersing cells by repeated pipetting. Cells were initially grown in RPMI 1640 containing glutamine (VWR, #95042-508), supplemented with 20% FBS (Thermo Fisher Scientific, #10437028), 5% penicillin–streptomycin solution (Sigma-Aldrich, P4333-100ML), 1% nonessential amino acids (Sigma-Aldrich, #TMS-001-C), and 1% sodium pyruvate solution (Sigma-Aldrich, #S8636-100ML). After 48 hours, media was aspirated and replaced with similar media containing only 2% penicillin–streptomycin solution. Media was then replaced every 3 to 4 days until the dish became 33% to 67% confluent. Cells were then trypsinized with Trypsin-EDTA (Sigma, #T3924-100ML) for 3 to 10 minutes until nonadherent, combined 1:1 with complete RPMI, and cell suspension was centrifuged at 1,000 *g* for 2 minutes. Cells were transferred to a new 6-well dish and allowed to confluence. Once confluency was reached, cells were transferred to larger plates for culturing and freezing aliquots in 10% DMSO. Once cell lines were established by growing in culture for a minimum of 2 weeks containing two trypsinization events, cells were grown as cell lines in complete RPMI. Complete RPMI consisted of 500 mL RPMI 1640 containing glutamine, 5 mL of sodium pyruvate solution, 5 mL of penicillin–streptomycin solution, and 50 mL FBS. Cells were passaged within a day of surpassing 80% confluency by 1:10 dilution. Cell lines from this study have been short tandem repeat–profiled by the ATCC Authentication Service, most recently in 3/13/24. Cells were tested for *Mycoplasma* and other pathogens prior to experimentation and freezing, by Charles River Research Animal Diagnostic Services Infectious Disease PCR, on 2/14/23. All cells were used at less than 10 passages from the original isolation or frozen tube.

### Transcriptome sequencing and comparative analysis

RNA was extracted from cells using a Qiagen RNeasy Miniprep kit (#R1054). RNA quality was assessed using Agilent 4200 TapeStation, and RINe values ranged from 8.6 to 10. Polyadenylated RNA was captured from 500 ng of total RNA per sample, and libraries were prepared using NEBNext Poly(A) mRNA Magnetic Isolation Module (#7490L) and NEBNext Ultra II Directional RNA Library Prep Kit for Illumina (NEBNext, #7760L). Paired-end sequencing was done at the Vanderbilt VANTAGE core laboratory (Vanderbilt University) to a depth of 25 million reads per library using Illumina NovaSeq 6000. Raw fastq.gz data were uploaded into Galaxy (21). GRCm38.86 (mm10) gtf.gz and toplevel.fa.gz reference files were obtained from Ensembl (RRID: SCR_002344; ref. 22). RNA STAR (23) was used to align paired-end 150 bp reads to mm10, followed by PCR duplicate removal by SAMtools RmDup (RRID:SCR_002105; ref. 24). Mapped reads were attributed to transcripts using featureCounts (25) and quantified for gene and transcript expression fragments per kilobase of transcript per million reads mapped (FPKM) using Cufflinks (26). Expression of CGT>CAT mutant p53 was manually verified in read data using mapped read visualization in the University of California Santa Cruz (UCSC) Genome Browser (RRID: SCR_005780) mm10 database around region chr11:69,589,594-69,589,622. One of eleven samples from an *ex vivo* cell line did not retain expression of the mutated copy of p53 (F361 tumor cells) despite having mapped RNA reads to this codon. FPKM values per sample were combined into transcripts per million reads tables using R to enable normalized sample-to-sample comparisons (27).

For human cancer cell lines to compare with, human Cancer Cell Line Encyclopedia (CCLE; 28) FPKM expression data were used and converted to TPM reads. To enable cross-species comparison, reciprocal ortholog data were utilized. Mousemine.org (29) and Humanmine.org (30) were used for ortholog identification, and the intersections of genes were used as reciprocal orthologous genes, resulting in 13,229 genes expressed and common orthologs between the two datasets. Human cell lines compared with mouse cancer cell lines included all lines annotated as from the cervix, endometrium, and ovary (*N* = 80 cell lines). Transcriptomes were pairwise compared using a Euclidean distance metric of all expressed genes. Nearest neighbor human cell lines comprise the cell lines shown. Gene expression in this figure was further normalized for visual comparison through mean transcription normalization, including the additional cell lines HELA, KURAMOCHI, SIHA, A2780, CAOV4, MDA-MB-231, MDA-MB-453, and KHYG (RRID: CVCL_0134, RRID: CVCL_0062, and RRID: CVCL_0418), chosen as representative cell lines which have mutational characteristics as meaningful to compare with the mouse cell lines (see “Results”). Shown signature genes were the top 25 genes with minimal expression differences within the mouse cell lines and nearest neighbor human cell line and maximal differences with non–nearest neighbor cell lines. Database for Annotation Visualization and Integrated Discovery (DAVID) (31) was used to generate the pathway annotations shown in the transcriptional signature.

### CNA analysis

Raw counts from featureCounts were input into InferCNV to assess CNAs. Settings for InferCNV included i3 copy-number variant calling, cutoff value of 1, Hidden Markov Model transition probability of 1e−02, and a window length of 300. Samples F326 splenic tumor, F326 ROV, and F361 tumor were used as control inputs due to lower levels of initial copy number variant calls in these samples.

### Syngeneic i.p. modeling

Cells, lentivirally labeled with GFP, were grown to 5 to 10 million per mouse and injected in 200 μL iced PBS via i.p. injection. Recipient mice were 3-month-old female OvTrpMyc mice. Mice were monitored for euthanasia criteria matching the longitudinal study. Tumors, uterine horns, attached fallopian tubes, ovaries, and fat pads were then immediately fixed and processed for IHC.

### Protein collection and immunoblotting of cell lines

Lysis of cells was performed with modified RIPA buffer (50 mmol/L Tris, 150 mmol/L NaCl, 1 mmol/L EDTA, 1% NP-40, and 1% sodium deoxycholate) with protease/phosphatase inhibitors (Thermo Fisher Scientific, #1861284) followed by sonication. SDS-PAGE was performed by standard methods, and antibodies were used for the detection of MYC (Abcam Cat# ab32072, RRID: AB_731658, 1:1,000 dilution) and GAPDH (Cell Signaling Technology Cat# 2118, RRID: AB_561053, 1:10,000 dilution). The secondary antibody was anti-rabbit IgG, horseradish peroxidase–linked (Cell Signaling Technology, Catalog #7074).

### Data availability

Next-generation sequencing raw and processed data are available via Gene Expression Omnibus (GEO) with accession number GSE238158.

## Results

### Generation of a human-mimetic genetic model of HGSOC and serous endometrial cancer

**Figure 1:**
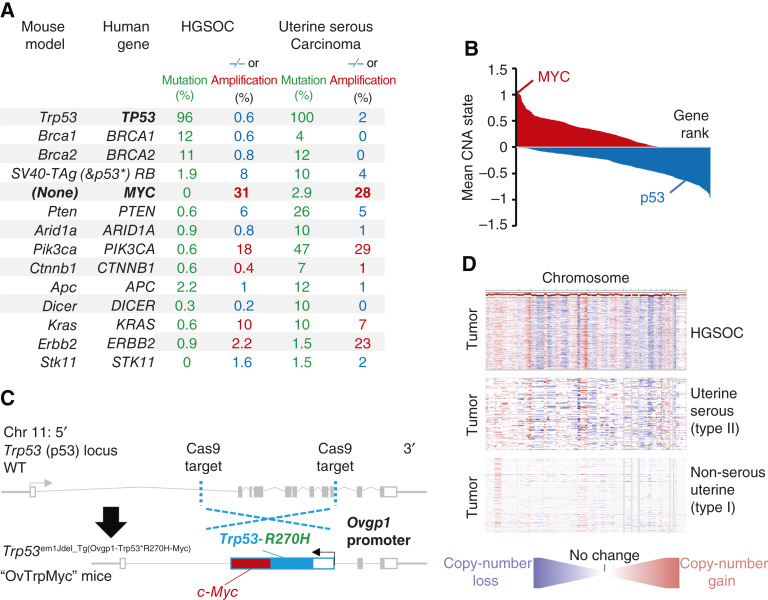
Rationale and design of the transgenic Ovgp1-Trp53-R270H-Myc mouse model. **A,** Comparison of currently available HGSOC mouse models genetics to genetic alterations in human HGSOC and serous endometrial cancers. −/− refers to homozygous deletion (blue, tumor suppressors), whereas Amp refers to copy-number amplification of at least two extra copies (red, oncogenes). **B,** Average copy-number state using TCGA HGSOC data at the gene level. Genes are plotted by ranking, with *MYC* and *TP53* highlighted. **C,** Design of the transgenic OvTrpMyc mouse model. The endogenous Trp53 allele was targeted using a spCas9 and homology donor repair template strategy in the C57BL/6Tc background. Transgene genotyped pups were backcrossed with C57BL/6J mice and heterozygous mice used in all studies. The *Ovgp1*-driven transgene includes a dominant-negative murine p53-R270H mutant sequence and a murine *c-Myc* sequence separated by a P2A self-cleaving peptide. **D,** Comparison of CNAs across the genome for TCGA studied HGSOC and UCEC patient tumors. Red indicates copy-number gain, whereas blue indicates copy-number loss. TCGA, The Cancer Genome Atlas.

Previous models of epithelial ovarian cancer have been used to seek a better understanding of the disease using known mutations. Ovarian cancer most commonly presents as HGSOC, which contains p53 mutation in at least 94% of cases (32, 33) and sporadic *BRCA1* or *BRCA2* mutations in <15% of cases. Germline *BRCA1* or *BRCA2* mutations yield ovarian cancer, most often HGSOC, with a lifetime unmitigated risk of 30% to 70% (34). Other mutations are present in 15% or less of patient primary tumors, including *APC*, *MUC16*, *ARID1A*, *RB1*, *NF1*, *CSMD3*, *CDK12*, *FAT3*, and *GABRA6* (32, 35). However, HGSOC exhibits unusually high levels of CNAs and aneuploidy, affecting two thirds of all genes. Low-grade serous carcinomas are driven by *BRAF* and *KRAS* and represent <5% of ovarian tumors (36). Mucinous tumors are driven by *KRAS* or *ERBB2* amplification, representing about 3% of ovarian tumors. Endometrioid and clear-cell carcinoma each represent about 10% of ovarian tumors and have high rates of *ARID1A* mutations (30% and 50%, respectively) and *PTEN* alterations, including deletions and mutations. Clear-cell carcinomas mutate *PIK3CA* in 33% of tumors. Mouse models of ovarian cancer have utilized these known mutations, which are reviewed by Howell (37) and Zakarya and colleagues (38). A comparison of these mouse driver genes to known human genetic driver frequencies is shown in Fig. 1A. Notably, uterine serous carcinoma, a type II endometrial cancer, exhibits very similar mutation patterns to HGSOC.

We sought to create a new genetic model, Trp53^em1Jdel_Tg(Ovgp1-Trp53*R270H-Myc)^, nicknamed OvTrpMyc, which accurately depicts *BRCA* and *PTEN* normal HGSOC. We and other researchers have found that the most commonly and excessively amplified region of the HGSOC genome is 8q24 (Fig. 1B; refs. 14, 32). Expression of a strong oncogene located in 8q24, *c-Myc*, was coupled with the expression of mutant p53. MYC is a basic helix-loop-helix leucine zipper transcription factor that regulates cell stemness, metabolism, and apoptosis and cell-cycle progression (39). Ovarian cancers require MYC for proliferation (40). The most common mutation of p53 in HGSOC (32) is a dominant-negative, gain-of-function (41, 42) amino acid mutation R273H, which maps to R270H in mice. We engineered mice via CRISPR-Cas9–targeting to insert a transgene driven by *Ovgp1* into the native *Trp53* (p53) locus of chromosome 11 (Fig. 1C). *Ovgp1* was previously demonstrated to express specifically in nonciliated fallopian fimbriae cells, not on the uterine epithelium and not on the OSE (11, 12). We confirmed FTE-selective staining patterns using the transgene’s HA peptide tag within the OvTrpMyc model (Supplementary Fig. S1B). However, the *Ovgp1* promoter used in this study to drive the transgene differs from those used in previous studies, and it is possible that off-target expression also differs. The transgene was inserted at the p53 locus to allow for CNAs to evolve, including on the p53 resident chromosome, as CNAs are characteristic of HGSOC. All transgenic mice in this study were genotyped as heterozygous for the transgene. HGSOC CNAs are similar to uterine serous carcinoma but not type I uterine cancer (Fig. 1D).

### Middle-age disease presentation

**Figure 2:**
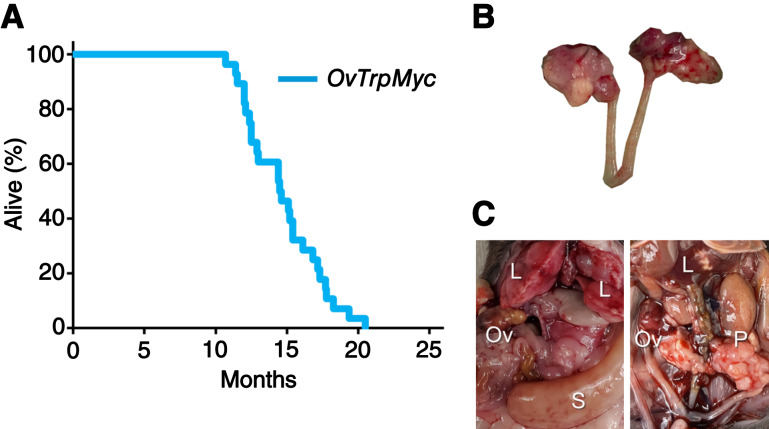
Middle-age presentation of ovarian and peritoneal tumors. **A,** Lifespan data from mice euthanized due to tumor burden criteria. **B,** Example of a dissected reproductive tract from a mouse with obvious macroscopic ovarian tumors. **C,** Examples of widespread intra-abdominal disease, including liver invasion (L), peritoneal tumors (P), splenic tumor (S), and ovarian tumors (Ov). L, liver invasion; Ov, ovarian tumors; P, peritoneal tumors; S, splenic tumor.

The median age of tumor burden–induced euthanasia for the OvTrpMyc mice was 14.5 months (Fig. 2A) or, given the limitations of human–mouse comparisons, about 40 to 60 years of human age [based on life expectancy of 80 years old, 896 days of female C567BL/6 mouse lifespan (43), with perimenopause in mice occurring around 9 months of age (44)].

OvTrpMyc mice were designed with limited oncogenic stimuli with the intent of long latency, consistent with human disease. However, OvTrpMyc mice are effectively *Trp53*^+/−^ in all cells. Heterozygous *Trp53*^+/−^ mice develop lymphomas, osteosarcomas, fibrosarcomas, hemangiosarcomas, lung adenocarcinomas, and other tumors, which form lethal tumors in 25% of mice requiring euthanasia as early as 16.5 months in the C57BL/6 background (20). Complete knockout *Trp53*^−/−^ mice develop primarily lymphomas and have a half-life of 4.5 months. We observed our OvTrpMyc mouse model to have a survival curve intermediate between these genotypes. *Pax8*-Cre–driven mixed-background models knocking out *Pten* and *Trp53*, in contrast, have a median age of euthanasia requirement between 3 and 6 months, depending on the full genotype (17).

### Pathology of lethal ovarian and uterine cancers

Gross examination of mice meeting euthanasia criteria showed HGSOC and other cancer phenotypes. For HGSOC, tumors of the ovary were occasionally observed (Fig. 2B), but large tumors were rare. Widespread intra-abdominal disease was occasionally observed (Fig. 2C), with tumors throughout the mesentery as well as the peritoneal wall. Tumors involved with muscle external to the abdomen were observed, typically with invasion spreading from the peritoneal wall or from the ovary.

**Figure 3:**
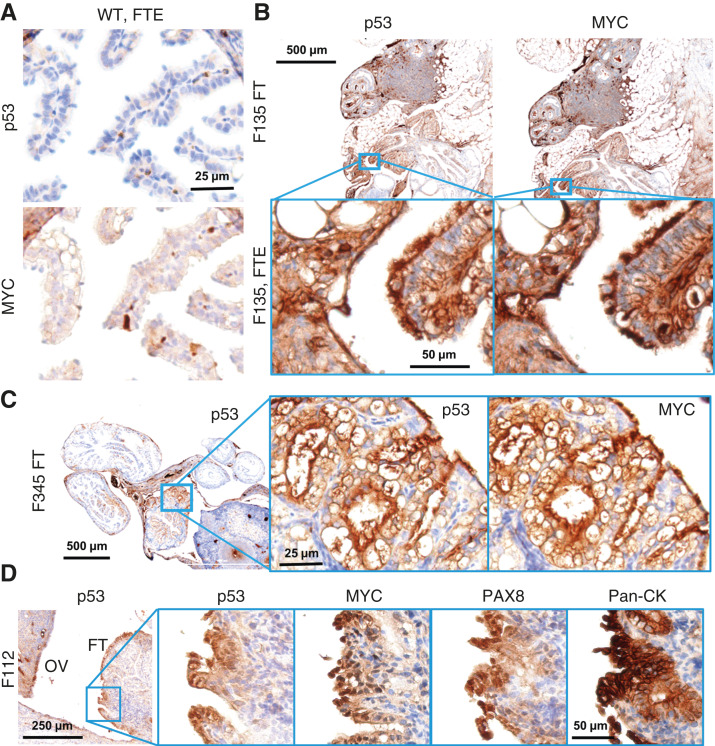
Molecular histology of the fallopian fimbriae. **A,** A WT mouse at 12 months of age showing little p53 or MYC staining in the FTE. **B,** OvTrpMyc fallopian tube at 15 months of age, F135, exhibiting p53 and MYC staining within the FTE. **C,** An OvTrpMyc mouse at 12 months of age, with vacuolated fallopian fimbriae–positive for p53 and MYC. **D,** An OvTrpMyc mouse at 15 months of age, with features resembling STIC.

Previous, carefully controlled studies demonstrated *Ovgp1* expresses on nonciliated epithelial cells within the FTE, not the OSE (11, 12). To investigate OvTrpMyc transgene protein products at end-stage disease, the gynecologic tract (including uteri, fallopian tubes, ovaries, and ovarian fat pads) was dissected from mice meeting euthanasia criteria at 10 to 16 months of age and processed for molecular pathology. Tissues were formalin-fixed and embedded in paraffin prior to sectioning and staining for p53 and MYC. Tumors, if observed, were additionally stained with paired-box transcription factor PAX8, a biomarker used to help define gynecologic tumors in humans (45–47) but is also expressed in embryogenesis of the thyroid, renal, and upper urinary tracts. Whereas background staining was observed, WT mice did not exhibit clusters of p53 or MYC positivity on the FTE, although scattered single cells were positive (Fig. 3A). In single sections, some FTEs from OvTrpMyc mice were also negative, like WT, perhaps because the entire fallopian tube was not examined due to limited material availability. However, 64% of mice requiring euthanasia exhibited positive FTE staining with p53 and MYC in the single section evaluated, which is likely an underestimate. This contrasts with young mice: in three of three 3-month-old OvTrpMyc mice, none showed positive staining despite clear transgene expression by 10 weeks of age (Supplementary Fig. S1B). Semiquantitative notes from all IHC-assessed mice are available in Table 1 for mice meeting humane euthanasia criteria and Supplementary Table S1 for healthy mice euthanized prior to lethal tumor burden.

**Table 1:** Semiquantitative estimates of IHC staining in moribund mice

Mouse ID	Genotype	Months	Euthanasia criteria met	Tissue	p53	MYC	PAX8
F129	Het	10.7	Y	FTE	0	0	NE
F129	Het	10.7	Y	OSE	4	4	1
F129	Het	10.7	Y	ULE	3	3	3
F259	Het	11.4	Y	FTE	1	1	NE
F259	Het	11.4	Y	OSE	0	0	0
F259	Het	11.4	Y	ULE	4	3	3
F345	Het	12	Y	FTE	3	3	NE
F345	Het	12	Y	OSE	0	0	0
F345	Het	12	Y	ULE	0	0	0
F376	Het	12.1	Y	FTE	0	0	NE
F376	Het	12.1	Y	OSE	0	0	0
F376	Het	12.1	Y	ULE	3	3	2
F127	Het	12.5	Y	FTE	1	1	—
F127	Het	12.5	Y	OSE	4	4	—
F127	Het	12.5	Y	ULE	3	3	4
F340	Het	12.9	Y	FTE	0	3	NE
F340	Het	12.9	Y	OSE	2	2	2
F340	Het	12.9	Y	ULE	0	0	0
F135	Het	14.6	Y	FTE	3	3	NE
F135	Het	14.6	Y	OSE	—	—	—
F135	Het	14.6	Y	Tumor-RP	4	4	4
F135	Het	14.6	Y	ULE	0	0	0
F2	Het	15.1	Y	FTE	—	—	—
F2	Het	15.1	Y	OSE	0	—	—
F2	Het	15.1	Y	Tumor-RP	4	4	4
F2	Het	15.1	Y	ULE	3	4	3
F283	Het	15.1	Y	FTE	0	3	NE
F283	Het	15.1	Y	OSE	—	—	—
F283	Het	15.1	Y	ULE	0	0	0
F112	Het	15.4	Y	FTE	4	4	NE
F112	Het	15.4	Y	OSE	4	4	4
F112	Het	15.4	Y	ULE	4	4	4
F278	Het	16.1	Y	FTE	1	1	NE
F278	Het	16.1	Y	OSE	3	3	2
F278	Het	16.1	Y	Tumor-M	4	4	4
F278	Het	16.1	Y	ULE	1	1	1
F12	Het	20.5	Y	FTE	1	2	NE
F12	Het	20.5	Y	OSE	0	0	0
F12	Het	20.5	Y	ULE	0	1	0

Abbreviations: M, mesenteric; RP, retroperitoneal.

p53, MYC, and PAX8 stains were enumerated: 0, no staining or scattered cells only; 1, clustered cells with predominantly cytoplasmic staining; 2, clustered cells with nuclear staining observed; 3, multiple clusters of cells with nuclear positivity; 4, majority of an area affected by positive staining.

“—” indicates tissue was not available or otherwise not evaluable on this slide. “NE” indicates PAX8 is unevaluable by this scoring on the FTE due to positivity in the normal, WT FTE.

Morphology and cellular distribution of p53 and MYC varied between mice. Mouse F135 exhibited nuclear p53 and nuclear MYC within the FTE, indicative of HGSOC, with surrounding cells exhibiting a mix of nuclear and cytoplasmic staining (Fig. 3B). This mouse had a large retroperitoneal tumor extending from the ovary to the spinal cord. The tumor stained positive for PAX8 in addition to p53 and MYC, as did a small mass situated in the ovarian fat pad (Supplementary Fig. S2), consistent with known HGSOC homing to adipocytes (48). Other mice exhibited FTE staining with primarily cytoplasmic expression surrounded by vacuoles (Fig. 3C). STIC is a precursor to HGSOC found in as many as 8% of FTEs of patients with *BRCA1/2* mutations from risk-reducing salpingo-oophorectomy and a higher proportion of patients with HGSOC (49). FTE from mouse F112 presented with features of STICs, including enlarged nuclei along the p53-positive epithelium, nuclear atypia, loss of polarity, and nearby morphologically normal regions (Fig. 3D). However, there were features which differ from human STICs, including occasional ciliated cell positivity and not strongly nuclear p53 in all affected cells, although MYC was strongly nuclear. Additional PAX8 and H&E images associated with p53 and MYC panels are provided as Supplementary Figs. S3 and S4.

**Figure 4:**
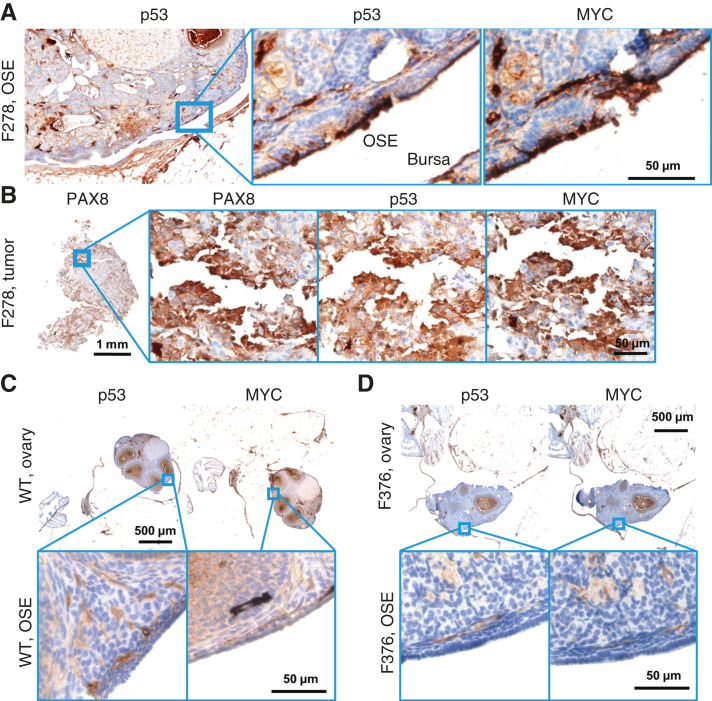
Molecular histology of the OSE. **A,** An OvTrpMyc mouse at 16 months of age with clear OSE staining of p53 and MYC. **B,** The intra-abdominal tumor extracted from the mouse shown in **A** exhibited PAX8, p53, and MYC staining. **C,** A WT mouse at 12 months of age is negative for p53 or MYC staining in OSE. **D,** Example of an OvTrpMyc mouse with a region of negative OSE staining of p53 and MYC, at 12 months of age.

OSE staining of p53 and MYC was observed in 50% of OvTrpMyc mice studied. In OvTrpMyc mouse F278, nuclear positivity of p53 and MYC was observed in cell clusters on the OSE (Fig. 4A), as was PAX8 (Supplementary Fig. S3). Mouse F278 exhibited minimal cytoplasmic positivity in the FTE and uterine luminal epithelium (ULE). The intra-abdominal tumor found in mouse F278 displayed PAX8 staining in addition to p53 and MYC (Fig. 4B). Other transgenic mice, similar to WT mice, were not observed to have p53 or MYC positivity on the OSE. Like WT mice, other ovarian structures stained positive (Fig. 4C and D). The OSE observations positive for p53/MYC may be intraepithelial metastasis events.

**Figure 5:**
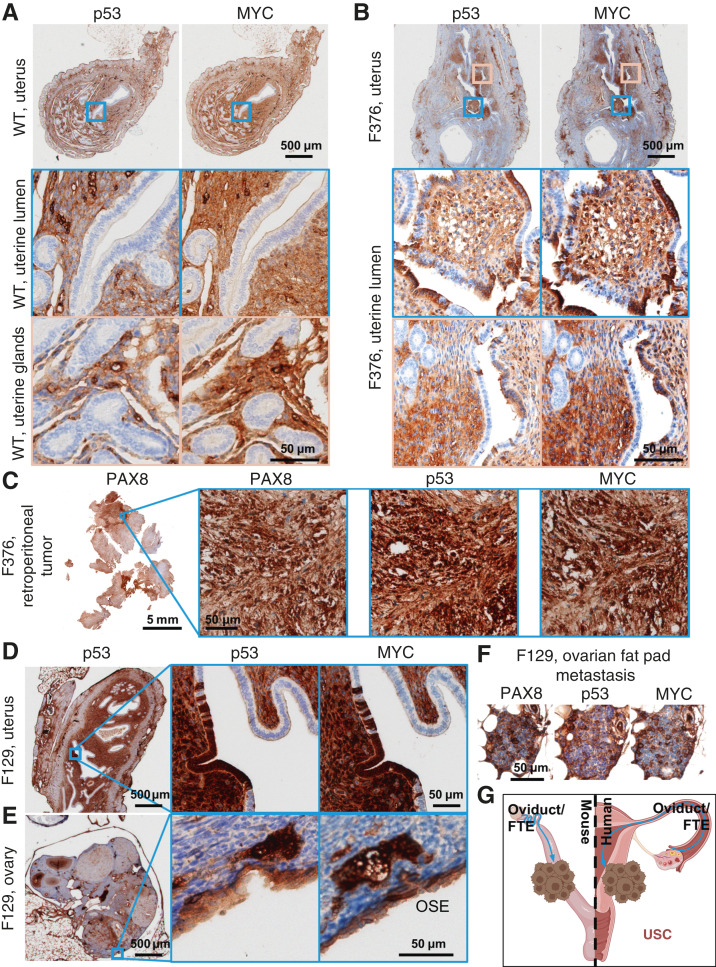
Uterine luminal epithelium staining of p53 and MYC. **A,** A WT mouse at 12 months of age showing negative p53 and MYC staining in the uterine epithelium and positive staining in adjacent tissues. **B,** Example of a 12 months old OvTrpMyc mouse with cytoplasmic p53 and whole-cell MYC staining along the ULE. **C,** A large retroperitoneal tumor, behind the kidney, stained for p53, MYC, and PAX8. **D,** An example of ULE expression in an OvTrpMyc mouse F129, with evidence of similar whole-cell staining of p53 and MYC on the (**E**) OSE and (**F**) ovarian fat pad metastasis. Mouse F129 was 11 months of age. **G,** Proposed model for observed dissemination of cells from the FTE to the ULE as an origin of uterine serous carcinoma. USC, uterine serous carcinoma.

Although the OvTrpMyc mice were originally designed to mimic HGSOC, strong staining of p53 and MYC on the ULE was noted in 58% of OvTrpMyc mice. Adjacent tissue exhibited p53 and MYC staining in normal, WT mice, whereas uterine epithelium was universally negative for staining (Fig. 5A). In OvTrpMyc mice, p53-, MYC-, and PAX8-positive epithelial clusters were observed, flanked by scattered cells evidently spreading into the adjacent epithelium, which was not observed in WT mice (Fig. 5A and B; Supplementary Fig. S3). This occurred in the absence of OSE (Fig. 4D) or FTE staining (Supplementary Fig. S3) in the single section from OvTrpMyc mouse F376 but coincided with macroscopic tumor staining (Fig. 5C). However, uterine lumen epithelial staining coincided with predominantly cytoplasmic p53 and MYC staining found in the OSE in one of the youngest mice euthanized, at 11 months (Fig. 5D and E). Ovarian fat pad metastatic cells were found and included p53, MYC, and PAX8 staining (Fig. 5F). These results are consistent with the model that the observed uterine p53/MYC-positive cells possibly originated on the FTE, as driven by *Ovgp1*, and migrated through the oviduct into the uterus (Fig. 5G).

### Observation of non-gynecologic cancers

Macroscopic tumors were not always immediately observed upon dissection, despite a mouse reaching euthanasia criteria. Three such mice were subjected to full necropsies, revealing brain tumors and lung tumors (Supplementary Fig. S5). Tumors resembling sarcomas or carcinosarcomas were also identified from macroscopic neoplasms (Supplementary Fig. S6). It is unclear if these are metastatic events, a form of uterine carcinosarcoma, accelerated background strain tumor formation due to disruption of *Trp53*, or another event.

### Aneuploidy and CNAs within OvTrpMyc tumor cells

**Figure 6:**
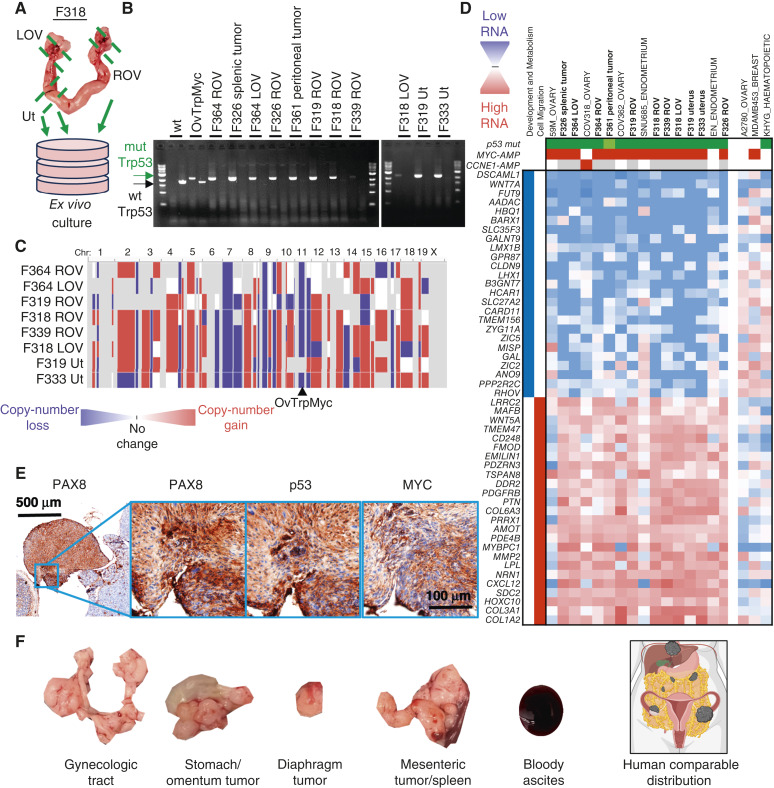
CNAs, transcriptomics, and tumorigenicity of extracted OvTrpMyc tumor cells. **A,** Dissection diagram for *ex vivo* expansion. Gynecologic tract for mouse F318 is shown. **B,** LOH was observed by PCR of the Trp53 transgene (mutation) in isolated cells. **C,** Whole-genome CNA calls of tumor cells. Blue indicates copy-number loss, and red indicates copy-number gain. **D,** Transcriptomic profiling of OvTrpMyc cancer cells compared with human gynecologic cancer within the human CCLE. Header genes refer to mutation in p53 (green), MYC amplification (red), or CCNE1 amplification (red), shaded white if absent or gray if unknown. Nearest neighbor human cell lines comprise the cell lines shown. A2780, MDA-MB-453, and KHYG cell lines are shown as representative cell lines which have driver gene characteristics comparable with the mouse cell lines but clearly different transcriptional profiles. **E,** 8 × 10^6^ F339 ROV OvTrpMyc cell line cells were injected i.p. into a young OvTrpMyc female recipient mouse. The mouse lost weight, meeting euthanasia criteria after 54 days. IHC staining of a dissected tumor attached to the intestines is shown. **F,** Distribution of tumors found in F318 LOV i.p. syngeneic injected mice (5 × 10^6^ cells, 132 days) relative to a human image depiction of HGSOC metastatic spread. LOV, left ovary; ROV, right ovary; Ut, uterus.

The primary rationale of integrating the OvTrpMyc transgene into the endogenous p53 locus was to allow for LOH of chromosome 11 (chromosome 17 in humans) during spontaneous tumor development. To test if LOH occurred in tumor cells from these mice, eight mice meeting euthanasia criteria were dissected (Fig. 6A) for small portions of the uterus, ovaries, and fallopian tubes and any macroscopic tumor presentation. These dissections were minced, washed, and allowed to grow *ex vivo* in tissue culture media (see “Materials and Methods”). Once a cell line was established with regular passaging, these cells were (i) genotyped for LOH and (ii) sequenced by bulk RNA sequencing for mRNA transcriptome analysis. Seven mice yielded a cell line from at least one of these origin sites. LOH was present in the *ex vivo* OvTrpMyc cell lines generated (Fig. 6B; mutant PCR bands were seen, but no WT bands were seen other than in controls). These results provide direct evidence that the WT allele of p53 was lost in these tumor cells. Each tumor cell line was shown to have ample chromosome alterations (Fig. 6C), consistent with patterns in human disease. Although synteny is partial for most chromosomes, loss of the p53-coding chromosome (11 in mice and 17 in humans) and gain of the mouse region syntenic to human amplicon 8q24, on murine 15qD1, was often found.

### OvTrpMyc tumor cells transcriptionally pair with HGSOC and uterine serous cancer

Transcriptomes from OvTrpMyc tumor cells were next analyzed to characterize which human gynecologic cancers they best mimic. Because cell lines have clear differences from endogenous disease (50), transcriptomes, we compared between mouse and human cell lines. Clustering of transcriptomes revealed that tumor cells from OvTrpMyc mice most closely mimicked well-characterized HGSOC and, unexpectedly, p53-mutant endometrial cancer cell lines but not cervical nor other subtypes of ovarian cancer (Fig. 6D). A singular exception in cell identity clustering was the EN cell line, which in the CCLE dataset, utilized for transcriptomes in Fig. 6D, was not annotated as p53-mutant nor *MYC*- or *CCNE1*-amplified. However, the founders of the EN line note a p53-R273H mutation (51), and the transcriptomic data from the CCLE (52) also show *MYC*, *CCNE1*, and p53 expressions as comparable with HGSOC lines. Cell lines with features that are only a portion of the transgene are shown for comparison. A2780 is a non-HGSOC ovarian cancer cell line but has neither a p53 mutation nor a MYC amplification. Triple-negative breast cancers have many similar characteristics from a genetic standpoint to HGSOC, including frequent BRCA mutation, p53 mutation, and high aneuploidy, but a representative cell line MDA-MB-453 displayed a clearly different transcriptomic profile. Lastly, the leukemia cell line KHYG, which has a p53 mutation, also displayed a clearly different transcriptomic profile. OvTrpMyc clusters revealed a downregulation of developmental and metabolic genes and an upregulation of cell migration genes. These transcriptome-wide data provide molecular evidence that complements histopathology to confirm HGSOC and serous endometrial cancers arise in the OvTrpMyc GEMM.

Cell lines were amplified and injected intraperitoneally into recipient young female OvTrpMyc mice. The mice were monitored for disease meeting the same euthanasia criteria as the spontaneous disease model. Mice required euthanasia due to ascites or distorted abdomen at days 40 (F319 uterus), 54 (F339 ROV), 65 (F326 ROV), and 132 (F318 LOV). Upon euthanasia, gross pathology observations were noted, the gynecologic tract was dissected, and tissues were processed for histopathologic analysis. Histopathologic examination of intra-abdominal tumors showed evidence of HGSOC, including nuclear atypia and pleomorphism, cellular budding, PAX8 staining, nuclear p53 staining, and nuclear MYC staining (Fig. 6E). OvTrpMyc cell line protein levels of MYC were intermediate between high-MYC and low-MYC human cell lines (Supplementary Fig. S7). Macroscopic dissemination was similar to human HGSOC for F318 LOV cells (Fig. 6F). These F339 ROV and F318 LOV cells are evidently useful for syngeneic injections for controlled experimentation about HGSOC tumor formation and development. The F319 uterus cell line exhibited rapid ascites and may be considered for uterine serous carcinoma syngeneic experiments.

## Discussion

Our study demonstrates that MYC expression and a *p53-R270H* mutation drives HGSOC in a mouse model of *Ovgp1* promoter–driven transgene expression. No *BRCA* or homologous repair gene was altered in the GEMM design. The single transgene is sufficient for experimentation without extensive breeding protocols. Latency is long, 14.5 months on average, allowing for the development of HGSOC characteristics such as peritoneal metastasis and extensive CNAs. The FTE was aberrant in most moribund mice, with features consistent with STICs observed. Histopathologic observations were made, which were consistent with intraepithelial metastases to the OSE and ULE. Cells grown *ex vivo* from the gynecologic tract from transgenic mice formed tumors following i.p. injections. This mouse model may be used to study aspects of spontaneous cancer development and for syngeneic injection experimentation.

At morbidity, 100% of the studied mice exhibited evidence of p53 and MYC positivity along epithelial clusters in at least one site within the FTE, OSE, and ULE. More than half of the mice exhibited positivity at multiple sites. However, the largest tumor observed in these mice tended to be metastases, not the ovary itself. Indeed, when tumor cells were isolated for *ex vivo* growth, the ovary and oviduct seemed within the reference range of macroscopic size, albeit somewhat morphologically different. We speculate that this may be due to high metastatic potential of these cells which have both *p53* mutation and MYC overexpression, consistent with the finding that syngeneic mouse injections yielded lethal tumor burden. Given that *Myc* is a Yamanaka factor enabling dedifferentiation and redifferentiation into many tissue sites (53), it may be unsurprising that metastases predominate in this GEMM.

The OvTrpMyc mice have a longer latency than other models with strong drivers. SV40 T-antigen MISIIR mice develop ovarian tumors with complete penetrance, usually within 4 to 6 months of age (54). With *Ovgp1*, SV40-driven mice develop histologic lesions in the epithelium of the fallopian tube as early as 6 weeks of age (55). Mice with *Pax8-Cre*–driven *PTEN* knockouts were lethal at 2.2 to 5.7 months, from disease originating in fallopian STICs (17). Similar to *Brca1*−/−*p53*−/−*Rb*−/−*Nrf1*+/− mice (12), OvTrpMyc mice often require a year or more to develop observable disease. We predicted latency is required to accumulate aneuploidy, which we previously observed was somewhat rare in SV40 T-antigen–driven disease (14). Aneuploidy is characteristic of HGSOC and uterine serous cancer (32, 56, 57). A handful of aneuploid events were observed previously in *Brca2*−/−*Pten*−/−*p53*^*R270H*/−^–driven tumors (17). Here, OvTrpMyc mice clearly utilized aneuploid mechanisms for tumor development processes, with strong positive selection for the mutant allele of p53 on chromosome 11. CNAs were ubiquitous in cell lines derived from OvTrpMyc.

Fallopian fimbriae as a likely origin site of ovarian cancer was first suggested from histopathology of prophylactic oophorectomy from women with germline BRCA mutations (58). GEMMs have now provided ample support for epithelium within the fallopian fimbriae as a site with characteristics of disease initiation (37, 38). However, *Ovgp1*-driven mouse models all included a *Brca1*+/− or *Brca1*−/− genotype along with other driver genes. There remains clinical debate about whether salpingectomies should be performed in patients of average risk, particularly opportunistic procedures for contraception (10, 59). The OvTrpMyc model presented here is the first HGSOC GEMM showing evidence of precursor lesions in the fallopian fimbriae epithelium using an *Ovgp1* promoter without *Brca* deletions. This is supportive of the thesis that patients without germline *BRCA* mutation may nonetheless benefit from salpingectomies to reduce ovarian cancer risk.

Limitations of this model are varied. Some of the nongynecologic tumors found in late-life euthanized mice were analogous to those found in *Trp53*+/− mice. Although we also found that gynecologic cancer cells could be isolated in 88% of cases of a dissected mouse at end-stage disease, metastatic modeling of HGSOC in this spontaneous model is difficult. PAX8 and other biomarker staining can aid in interpretation of the site of origin. Mice envelop the ovary with a layer of tissue forming the bursa, which humans do not have, and this may affect metastatic properties. The bursa is additionally known to develop leiomyosarcomas in response to intrabursal viral inactivation of *Trp53* and *Brca1* (60). The IHC staining presented here contained significant background, which, if removed through optimization, would improve the understanding of tissue expression specificity and nuclear/cytoplasmic ratios. The mice did not always exhibit obvious macroscopic tumors, including on the ovaries, even in mice which clearly had viable cancer cells once dissected from the ovary (Fig. 6A), and ultimately formed tumors in a syngeneic setting. Lastly, although the *Ovgp1*-driven transgene product HA expressed primarily in the FTE (Supplementary Fig. S1), it is possible that some off-target expression occurs during the lifespan of a mouse.

The unexpected, yet exciting, finding that hallmarks of uterine serous carcinomas, such as cells resembling endometrial intraepithelial carcinoma, are found in the OvTrpMyc model provides further support that uterine serous cancer is similar genetically and biologically to HGSOC. Like HGSOC, the most common p53 alteration in uterine serous carcinomas is a mutation in R273 (murine R270). For CNAs, 8q24, containing *MYC*, is the second most common amplification just behind the unstable *MECOM* locus near *PIK3CA* (56). Previous serous endometrial GEMMs required loss of *PTEN* in a p53 null background or a mutation in a telomere shelterin complex gene to yield disease within the lifetime of a mouse (61, 62). Uterine serous carcinoma is aggressive and treatment-resistant like HGSOC; it only accounts for 10% of endometrial cancer cases, yet it is responsible for 39% of endometrial cancer–related deaths (63). Retrospective studies have revealed a link between tubal ligation, which may prevent intraepithelial metastasis from the fallopian tube, and reduced rates of endometrial cancer (64). In a carefully controlled cohort study of tubal ligation, the hazard ratio for type I endometrioid uterine cancer was 0.78, but the HR was strikingly 0.25 for type II, typically p53-mutant uterine serous carcinoma, indicating surprisingly low incidence of type II tumors following tubal ligation (65). Fallopian STICs and other fallopian epithelium irregularities were observed in patients with uterine serous carcinoma and included the same clonal p53 mutation (66, 67). Taken together, these human retrospective studies, the current OvTrpMyc GEMM study, and human genetic data (68, 69) are all consistent with the emerging hypothesis that uterine serous carcinoma can originate from the same site as HGSOC: the FTE.

## Supplementary Material

Supplementary Figure 1 Ovgp1 transgene expression

Supplementary Figure 2 Tumor immunohistochemistry from FTE affected mice

Supplementary Figure 3 PAX8 staining corresponding to other immunohistochemical figures

Supplementary Figure 4 H&E staining corresponding to immunohistochemical figures

Supplementary Figure 5 Necropsy of mice lacking macroscopic disease

Supplementary Figure 6 Other malignancies

Supplementary Figure 7 MYC levels in syngeneic OvTrpMyc cell lines relative to human lines

Supplementary Table 1 Semi-quantitative estimates of immunohistochemical staining in healthy mice
