# Supplementary material for: MYC is Sufficient to Generate Mid-Life High-Grade Serous Ovarian and Uterine Serous Carcinomas in a p53-R270H Mouse Model
Source: Cancer Res Commun. 2024 Sep 26;4(9):2525–38. doi: 10.1158/2767-9764.CRC-24-0144 (PMC11425777; doi:10.1158/2767-9764.CRC-24-0144)
Supplement: Supplementary Figure 1 — Ovgp1 transgene expression [file crc-24-0144_supplementary_figure_1_supps1.pdf]

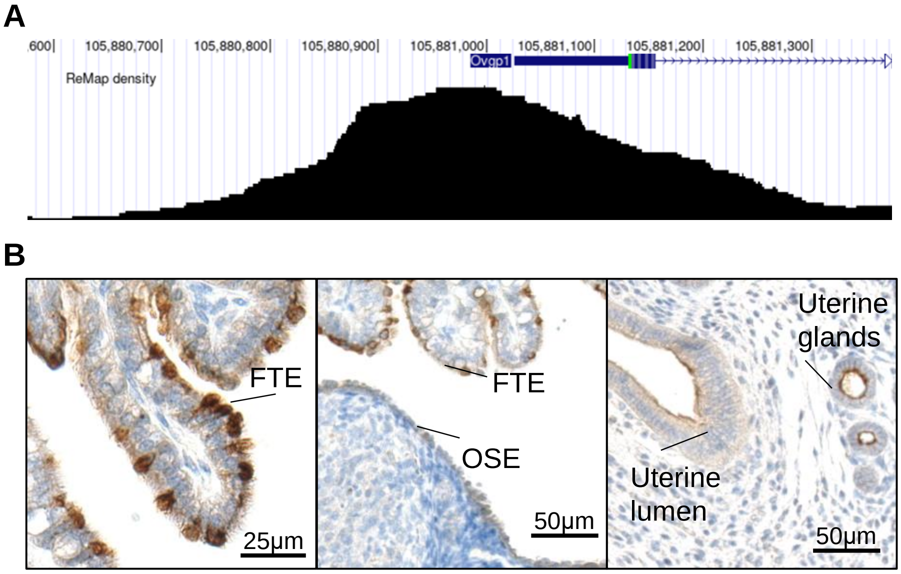

Figure S1: *Ovgp1* transgene expression. (A) UCSC Genome Browser display of the murine *Ovgp1* promoter with its regulatory peak, as annotated in the ReMap ChIP-seq database. (B) 3xHA tag transgene immunohistochemical staining at 10 weeks of age in an OvTrpMyc female.
