# Supplementary material for: MYC is Sufficient to Generate Mid-Life High-Grade Serous Ovarian and Uterine Serous Carcinomas in a p53-R270H Mouse Model
Source: Cancer Res Commun. 2024 Sep 26;4(9):2525–38. doi: 10.1158/2767-9764.CRC-24-0144 (PMC11425777; doi:10.1158/2767-9764.CRC-24-0144)
Supplement: Supplementary Figure 2 — Tumor immunohistochemistry from FTE affected mice [file crc-24-0144_supplementary_figure_2_supps2.pdf]

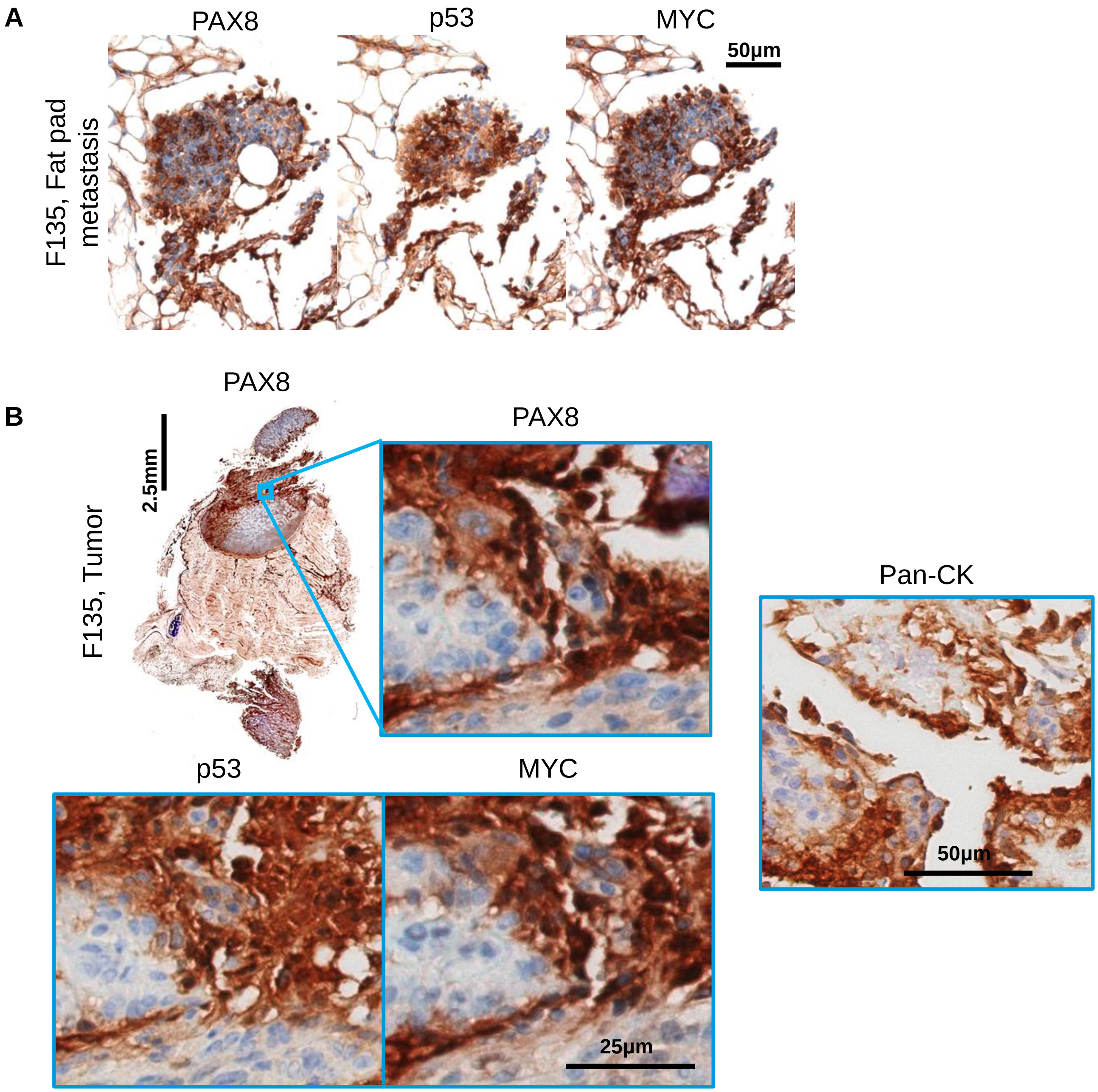

Figure S2: Tumor immunohistochemistry from FTE affected mice. (A) An ovarian fat pad metastatic nodule. (B) Intra-abdominal tumor dissected from an OvTrpMyc mouse with positive PAX8, p53, and MYC staining.
