# Supplementary material for: MYC is Sufficient to Generate Mid-Life High-Grade Serous Ovarian and Uterine Serous Carcinomas in a p53-R270H Mouse Model
Source: Cancer Res Commun. 2024 Sep 26;4(9):2525–38. doi: 10.1158/2767-9764.CRC-24-0144 (PMC11425777; doi:10.1158/2767-9764.CRC-24-0144)
Supplement: Supplementary Figure 3 — PAX8 staining corresponding to other immunohistochemical figures [file crc-24-0144_supplementary_figure_3_supps3.pdf]

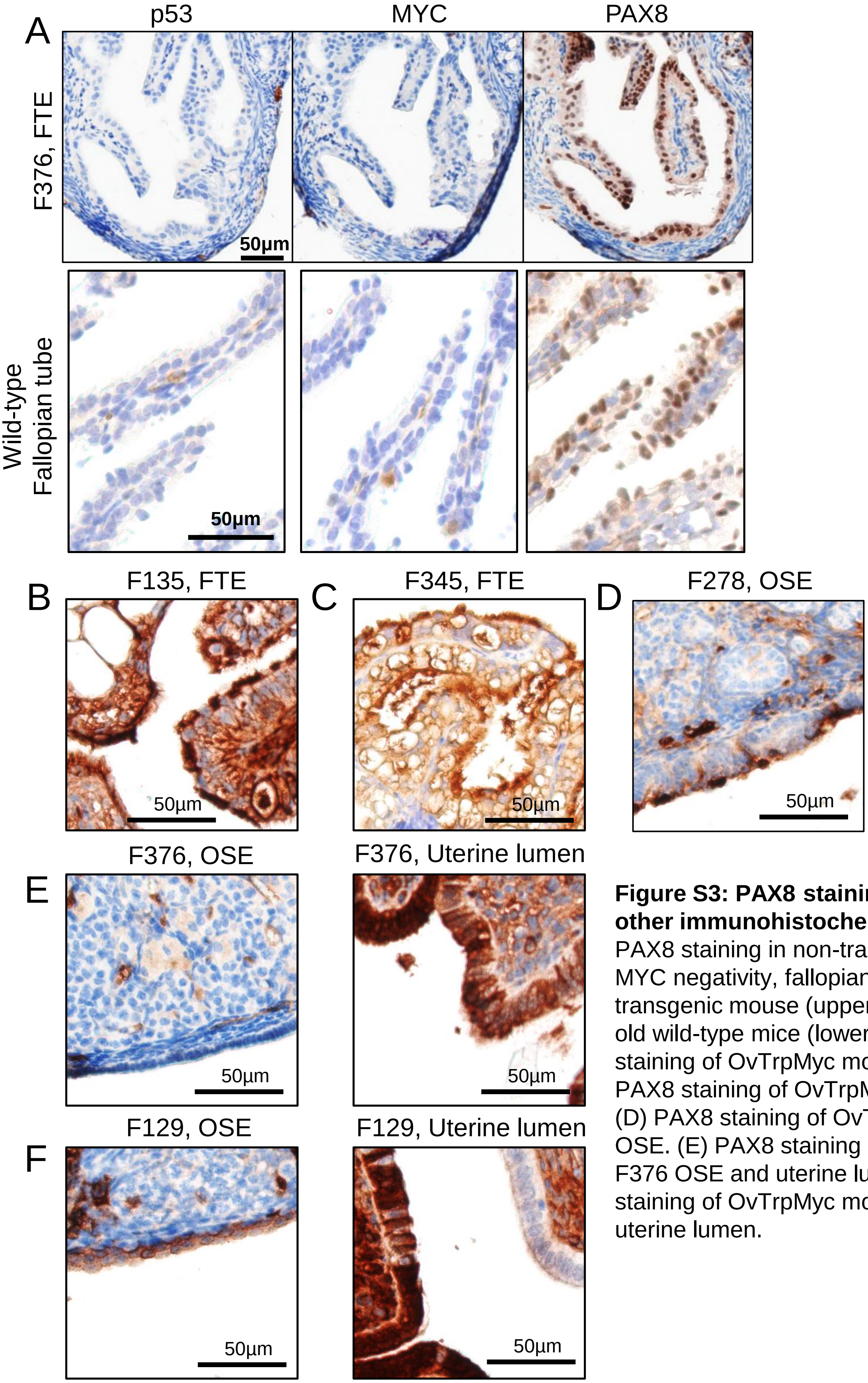

Figure S3: PAX8 staining corresponding to other immunohistochemical figures. (A) PAX8 staining in non-transformed, by p53 and MYC negativity, fallopian tube epithelium of a transgenic mouse (upper row) or in 12-month old wild-type mice (lower row). (B) PAX8 staining of OvTrpMyc mouse F135 FTE. (C) PAX8 staining of OvTrpMyc mouse F345 FTE. (D) PAX8 staining of OvTrpMyc mouse F278 OSE. (E) PAX8 staining of OvTrpMyc mouse F376 OSE and uterine lumen. (F) PAX8 staining of OvTrpMyc mouse F129 OSE and uterine lumen.
