# Supplementary material for: MYC is Sufficient to Generate Mid-Life High-Grade Serous Ovarian and Uterine Serous Carcinomas in a p53-R270H Mouse Model
Source: Cancer Res Commun. 2024 Sep 26;4(9):2525–38. doi: 10.1158/2767-9764.CRC-24-0144 (PMC11425777; doi:10.1158/2767-9764.CRC-24-0144)
Supplement: Supplementary Figure 4 — H&E staining corresponding to immunohistochemical figures [file crc-24-0144_supplementary_figure_4_supps4.pdf]

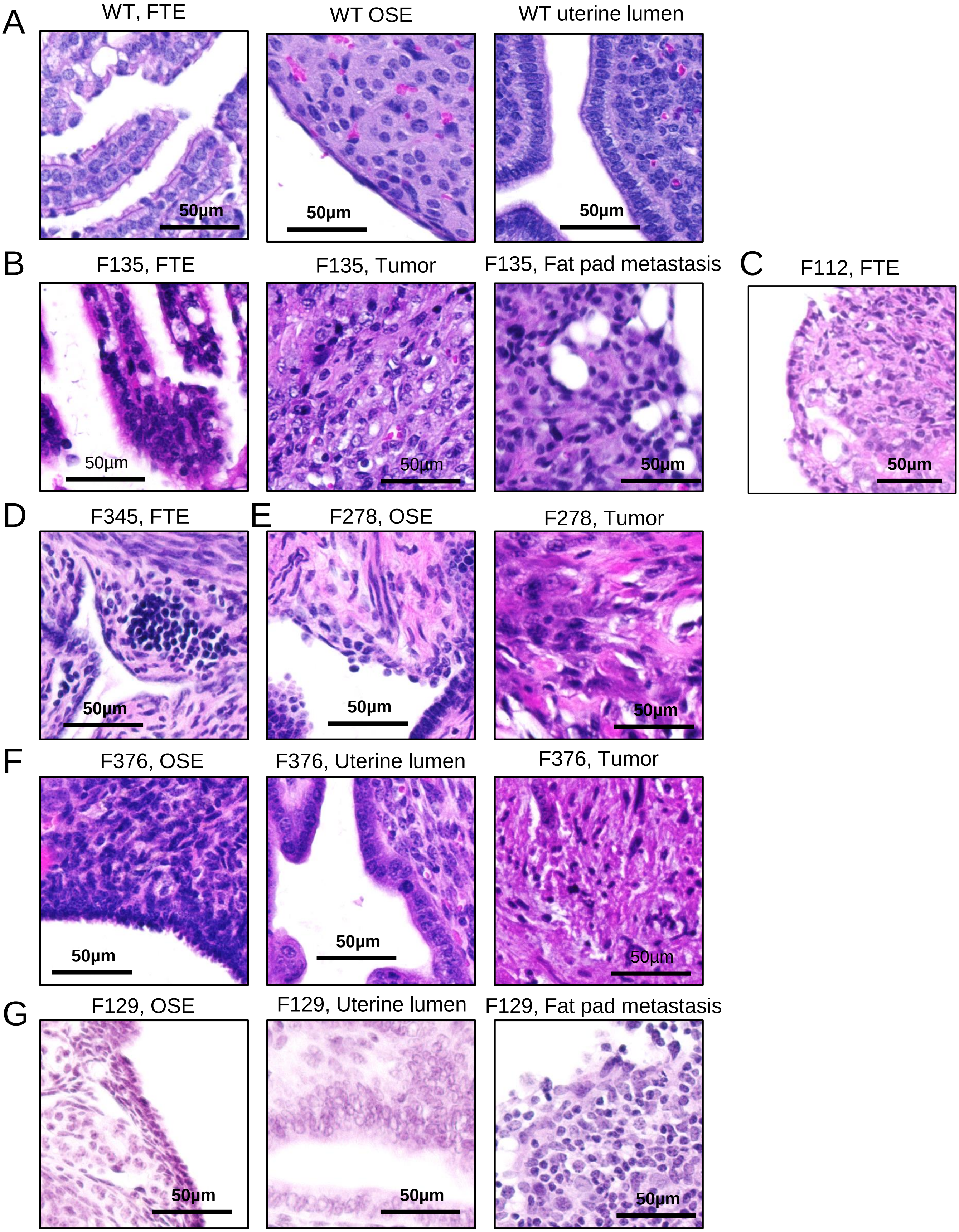

Figure S4: H&E staining corresponding to immunohistochemical figures. H&E staining was used in all images here. (A) WT mouse FTE, OSE, and uterine lumen (B) OvTrpMyc mouse F135 FTE, fat pad metastasis, and tumor. (C) OvTrpMyc F112 FTE. (D) OvTrpMyc mouse F345 FTE. (E) OvTrpMyc mouse OSE and tumor. (E) OvTrpMyc mouse F376 OSE, uterine lumen, and tumor. (F) OvTrpMyc mouse F129 OSE, uterine lumen, and fat pad metastasis.
