# Supplementary material for: MYC is Sufficient to Generate Mid-Life High-Grade Serous Ovarian and Uterine Serous Carcinomas in a p53-R270H Mouse Model
Source: Cancer Res Commun. 2024 Sep 26;4(9):2525–38. doi: 10.1158/2767-9764.CRC-24-0144 (PMC11425777; doi:10.1158/2767-9764.CRC-24-0144)
Supplement: Supplementary Figure 5 — Necropsy of mice lacking macroscopic disease [file crc-24-0144_supplementary_figure_5_supps5.pdf]

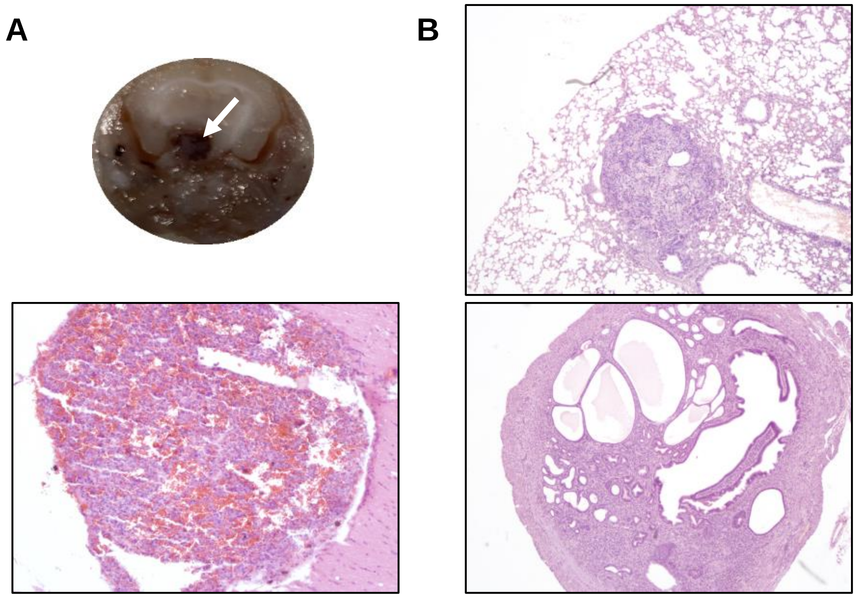

Figure S5: Necropsy of mice lacking macroscopic disease. OvTrpMyc mouse were evaluated by necropsy when macroscopic tumors were not immediately observed. **(A)** Dissected brain (top panel) and H&E section of brain tumor (bottom panel). **(B)** Lung metastasis (top panel) and a normal phenotype of some aged mice: cystic uterus (bottom panel).
