# Supplementary material for: MYC is Sufficient to Generate Mid-Life High-Grade Serous Ovarian and Uterine Serous Carcinomas in a p53-R270H Mouse Model
Source: Cancer Res Commun. 2024 Sep 26;4(9):2525–38. doi: 10.1158/2767-9764.CRC-24-0144 (PMC11425777; doi:10.1158/2767-9764.CRC-24-0144)
Supplement: Supplementary Figure 6 — Other malignancies [file crc-24-0144_supplementary_figure_6_supps6.pdf]

A

B

C

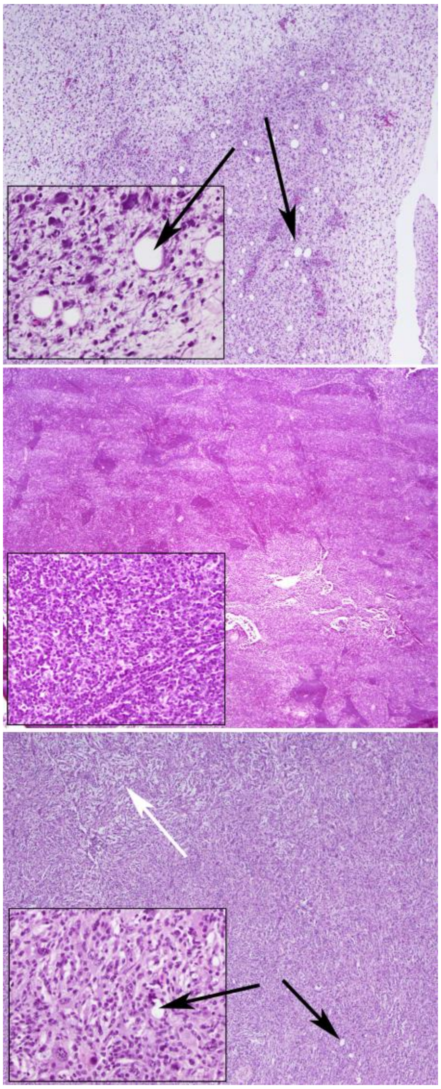

Figure S6: Other malignancies. (**A**) Myxoid neoplasm with markedly pleomorphic cells with associated vacuoles, marked by the black arrows. (**B**) Small round blue cell malignancy with relatively uniform nuclei growing in a sheeted growth pattern. (**C**) Tumor characteristics include spindle cell malignancy with scattered giant cells, storiform architecture, marked by the white arrow. Moderately pleomorphic with scattered vacuoles, marked by black arrows.
