# Supplementary material for: MYC is Sufficient to Generate Mid-Life High-Grade Serous Ovarian and Uterine Serous Carcinomas in a p53-R270H Mouse Model
Source: Cancer Res Commun. 2024 Sep 26;4(9):2525–38. doi: 10.1158/2767-9764.CRC-24-0144 (PMC11425777; doi:10.1158/2767-9764.CRC-24-0144)
Supplement: Supplementary Figure 7 — MYC levels in syngeneic OvTrpMyc cell lines relative to human lines [file crc-24-0144_supplementary_figure_7_supps7.pdf]

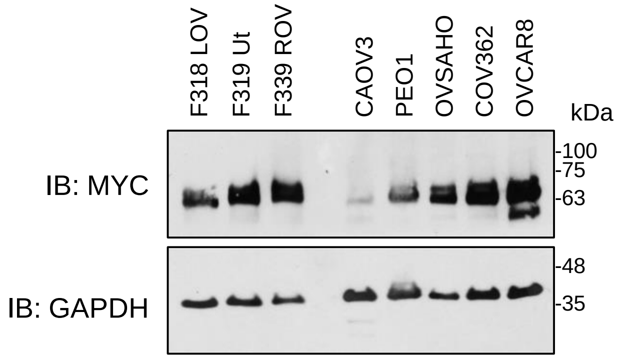

Figure S7: MYC levels in syngeneic OvTrpMyc cell lines relative to human lines. Western immunoblots (IB) of indicated cell line lysates.
