# Supplementary material for: MYC is Sufficient to Generate Mid-Life High-Grade Serous Ovarian and Uterine Serous Carcinomas in a p53-R270H Mouse Model
Source: Cancer Res Commun. 2024 Sep 26;4(9):2525–38. doi: 10.1158/2767-9764.CRC-24-0144 (PMC11425777; doi:10.1158/2767-9764.CRC-24-0144)
Supplement: Supplementary Table 1 — Semi-quantitative estimates of immunohistochemical staining in healthy mice [file crc-24-0144_supplementary_table_1_suppst1.pdf]

Mouse ID	Genotype	Months	Euthanasia criteria met	Tissue	p53	MYC	PAX8
F142	WT	3	N	OSE	0	0	0
F142	WT	3	N	FTE	0	0	NE
F142	WT	3	N	ULE	0	0	0
F28	WT	12	N	OSE	0	0	0
F28	WT	12	N	FTE	0	0	NE
F28	WT	12	N	ULE	0	0	0
FO111	Het	3	N	OSE	0	0	0
FO111	Het	3	N	FTE	0	0	NE
FO111	Het	3	N	ULE	0	0	0
FO112	Het	3	N	OSE	0	0	0
FO112	Het	3	N	FTE	0	0	NE
FO112	Het	3	N	ULE	0	0	0
FO113	Het	3	N	OSE	0	0	0
FO113	Het	3	N	FTE	0	0	NE
FO113	Het	3	N	ULE	0	0	0
FO86	Het	6	N	OSE	0	0	0
FO86	Het	6	N	FTE	0	0	NE
FO86	Het	6	N	ULE	0	0	0
FO67	Het	9	N	OSE	0	0	0
FO67	Het	9	N	FTE	1	1	NE
FO67	Het	9	N	ULE	0	0	0

Supplementary Table 1. Semi-quantitative estimates of immunohistochemical staining in healthy mice.
Tissue acronyms: fallopian tube epithelium (FTE), ovarian surface epithelium (OSE), uterine luminal epithelium (ULE).
p53, MYC, and PAX8 stains were enumerated 0: no staining or scattered cells only, 1: clustered cells with predominantly cytoplasmic staining, 2: clustered cells with nuclear staining observed, 3: multiple clusters of nuclear positive cells, 4: majority of an area affected by positive staining.
"-" indicates tissue was not available or otherwise not evaluable on this slide.
"NE" indicates PAX8 is unevaluable by this scoring on FTE due to positivity in normal epithelium.
